# Effects of dietary nitrate supplementation on isometric performance and physiological responses in college bodybuilders: a randomized, double-blind, crossover study

**DOI:** 10.3389/fnut.2025.1576712

**Published:** 2025-05-08

**Authors:** Lijun Wang, Ruohan Zhao, Yuhang Yan, Hongli Zhang, Ruixin Yan, Yifan Zhu, Zhuohong Han, Yue Qu, Ruonan Wang, Yicheng Li, Ruolin Chao

**Affiliations:** ^1^Institute of Physical Education, Shaanxi Normal University, Xi’an, China; ^2^Municipal Environmental Engineering Institute, Xian University of Architecture and Technology, Xi’an, China; ^3^College of Education, Beijing Sport University, Beijing, China; ^4^Xi’an Microelectronic Technology Institute, Xi’an, China

**Keywords:** beetroot juice, nitrate, isometric exercise, endurance, bodybuilder

## Abstract

**Introduction:**

In bodybuilding competitions, athletes are required to hold static poses for extended periods. This study aimed to evaluate the effects of acute beetroot juice (BJ) supplementation on isometric muscle endurance in college bodybuilding athletes.

**Methods:**

Sixteen male college bodybuilding athletes participated in a randomized, double-blind, crossover study conducted over three weeks with four laboratory visits. The first visit involved explaining the experimental protocol and performing the maximal voluntary isometric contraction (MVIC) test. The second visit familiarized participants with the testing procedures. During subsequent visits, participants consumed either BJ (250 ml,∼ 12.48 mmol of NO3−) or PL (250 ml,∼ 0.0005 mmol of NO3−), and blood samples were collected before testing to measure nitrate (NO3−) and nitrite (NO2−) concentrations. Participants then performed three rounds of isometric circuit endurance tests (ICET), during which heart rate (HR), ratings of perceived exertion (RPE), and blood lactate levels were recorded. Each round of ICET consisted of four subtests targeting the elbow flexors, core muscles, forearm muscles, and knee extensors, maintaining 70% of MVIC until fatigue. Additionally, surface electromyography (sEMG) was used to record and analyze muscle activity.

**Results:**

Compared to PL, acute BJ supplementation resulted in a 10.87-fold and 1.57-fold increase in serum NO3− and NO2− levels, respectively (*P* < 0.001). No significant differences were observed in MVIC peak torque under different conditions (*P* > 0.05). In the third round of testing (ICET-3), endurance improved by 14.9, 25.4, and 25.2% for the elbow flexors, forearm muscles, and knee extensors, respectively. No significant differences in root mean square (RMS) values were observed between the BJ and PL groups (*P* > 0.05).

**Discussion:**

These data suggest that acute beetroot juice supplementation had no significant effect on MVIC in college bodybuilding athletes but improved endurance in certain muscle groups during ICET. This suggests that nitrates may enhance endurance by optimizing intermittent recovery processes rather than directly increasing strength.

## 1 Introduction

Bodybuilding is a sport that places exceptionally high demands on muscle mass, definition, and overall esthetics. On stage, judges evaluate athletes’ muscle mass, symmetry, and definition based on a series of static bodybuilding poses (1). Repeated isometric contractions of different muscle groups are a hallmark of bodybuilding competitions. These poses require high-intensity isometric contractions, where athletes must maintain static positions with maximum muscle tension to highlight definition and contours. Competitors often endure intense flexing under hot stage lights for hours (2). From a physical perspective, achieving success in bodybuilding competitions requires exceptional isometric muscle endurance. Bodybuilders dedicate most of their competition preparation to the bulking phase (2). Consequently, most studies on bodybuilding supplements focus on their effects on training outcomes, such as resistance performance or muscle hypertrophy (3–6), while largely overlooking the static posing required during competitions. Previous studies have shown that during prolonged static contractions, an increase in intramuscular pressure leads to a significant reduction in muscle blood flow (7, 8). Restricted blood flow is a key factor limiting isometric endurance, as it directly impacts oxygen delivery to the muscle and restricts the production of adenosine triphosphate (ATP) during oxidative phosphorylation (9). It also indirectly exacerbates the development of muscle fatigue and reduces the clearance efficiency of metabolic waste products, such as lactate, carbon dioxide and reactive oxygen species (10, 11).

Beetroot, a natural source of nitrate (NO3−), has been extensively studied due to its high nitrate content, which serves as a precursor to nitric oxide (NO). After NO3− ingestion, facultative anaerobic bacteria on the dorsal surface of the tongue convert NO3− into bioactive nitrite (NO2−), which is further reduced to NO in the gastrointestinal tract and enters systemic circulation. The acidic and hypoxic conditions commonly occurring during exercise facilitate the conversion of NO2− into NO (12). NO promotes relaxation and subsequent dilation of vascular smooth muscle, enhances muscle oxygen perfusion, and facilitates gas exchange (13, 14). It has been shown to reduce blood pressure in skeletal muscles during exercise and mitigate the hypertensive response associated with isometric exercise (15, 16). Additionally, beetroot juice has garnered attention as a hypotensive agent, which may be particularly beneficial for bodybuilders, as studies indicate that 19.3% of males in this population exhibit hypertension (17). Interestingly, a recent meta-analysis revealed that while blood pressure responses in healthy adults are not significantly modified during exercise under nitrate supplementation (18), patients with arterial hypertension may experience beneficial, albeit limited, effects on these parameters (19). NO also enhances force and power generation during muscle contraction, reduces ATP demand, and decreases oxygen requirements for ATP synthesis (20). Research has shown that nitrate-rich beetroot juice supplementation exhibits inconsistent ergogenic effects across muscle groups during resistance exercise, with enhanced upper-body performance observed in movements like bench press (21, 22), while lower-body outcomes remain equivocal (23, 24). Additionally, evidence suggests its benefits extend to intermittent muscle function and recovery during repeated sprint exercises (25).

In summary, nitric oxide-mediated vasodilation in resistance exercise facilitates the delivery of oxygen and nutrients to active muscle tissues and replenishes ATP during rest intervals between repeated resistance bouts, potentially delaying fatigue and allowing for greater training volume (26, 27). Consequently, we hypothesize that BJ supplementation will improve neuromuscular performance across different muscle groups during isometric endurance testing and accelerate recovery during rest intervals.

## 2 Materials and methods

### 2.1 Participants

**FIGURE 1:**
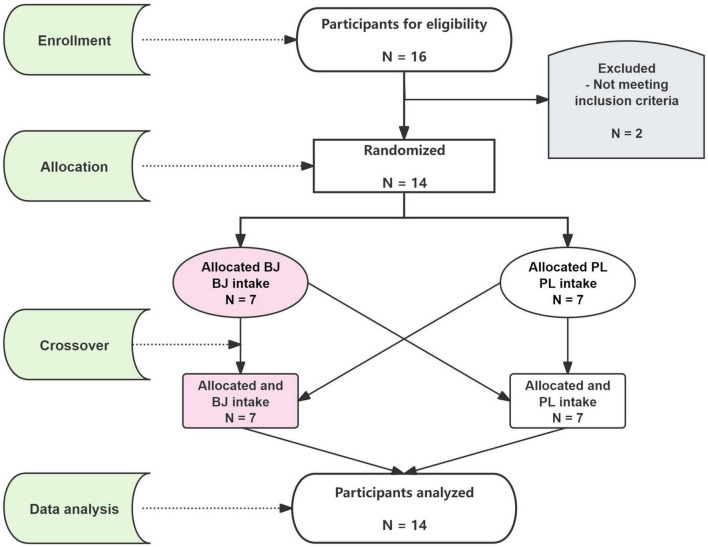
CONSORT flowchart of randomized, double-blind, crossover design. BJ, beetroot juice; PL, placebo.

The sample size for this study was determined using the effect size from a previously published study and calculated with G*Power software (version 3.1.9.4, University of Düsseldorf, Germany). Sample size calculation was based on a study by Husmann et al. (28) who determined the effects of beetroot juice vs. placebo supplement on exercise tolerance. The sample size was calculated using a medium standardized effect size of 0.3. A f-test family with repeated measures within factors, a power of 0.8, and alpha set at 0.05 indicated that 14 participants would be required. Participants were recruited from the fitness and bodybuilding varsity athletes of Shaanxi Normal University using a convenience sampling method. Sixteen male college bodybuilders (age: 23 ± 2 years; height: 180 ± 3 cm; weight: 84 ± 5 kg; training history: 5.3 ± 1.1 years; body fat percentage: 13.8 ± 2.3%; mean ± SD) voluntarily participated in this study. Figure 1 shows the CONSORT flowchart.

Inclusion criteria were as follows: (a) male college students with at least three years of resistance training experience; (b) participation in at least one regional or higher-level collegiate bodybuilding competition within the past year with a top-three placement in their group; (c) non-smokers, non-drinkers, and free from drug abuse. Exclusion criteria included exercise contraindications, cardiometabolic diseases, and the use of dietary supplements containing caffeine, sodium bicarbonate, creatine, β-alanine, and/or NO precursors (e.g., NO3−, arginine, citrulline, antioxidants). Given the potential sex-specific differences in physiological responses to nitrate intake, female participants were excluded (29). Participants were instructed to abstain from intense exercise, caffeine, dietary supplements, alcohol, and nitrate-rich foods for 24 h prior to their laboratory visits. A detailed list of nitrate-rich foods (e.g., beetroot, celery, lettuce, radish, spinach) was provided in advance. Additionally, participants were instructed not to brush their teeth, use mouthwash, or chew gum on the day of their laboratory visit. Finally, participants were asked to record their dietary intake 24 h prior to their first laboratory visit and replicate this intake 24 h before each subsequent visit. All participants were fully informed about the nature and purpose of the study and provided written consent to participate. All experimental procedures adhered to the ethical standards of the Declaration of Helsinki and were approved by the Research Ethics Committee of Shaanxi Normal University (code number: 202416059).

### 2.2 Experimental design

**FIGURE 2:**
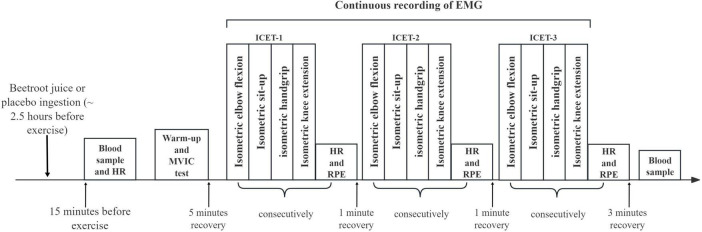
A schematic of the exercise protocol performed on visit 3 and 4 (i.e., experimental visits).

Participants were required to visit the laboratory four times over three weeks. During the first visit, the experimental procedures were explained to the participants, and a standardized maximum isometric strength test was conducted at specific joint angles to determine the resistance to be applied in subsequent visits. During the second visit, participants were familiarized with the Isometric Circuit Endurance Test (ICET; see below) and received technical instructions. During subsequent visits, participants arrived at the laboratory and remained seated with uncrossed legs for 5 min to achieve a resting state (30). Heart rate (HR) and blood lactate levels were then measured. Participants then performed three rounds of the ICET (ICET-1, ICET-2 and ICET-3) with one-minute intervals. HR and ratings of perceived exertion (RPE) were measured immediately after each ICET round, and blood lactate levels were measured again 3 min after the test. The protocol is illustrated in Figure 2. Additionally, surface electromyography (sEMG) was recorded throughout the measurement trials. During the third and fourth visits, conducted under a double-blind, randomized, crossover design, participants were assigned to two experimental conditions: consuming nitrate-rich beetroot juice (BJ) or nitrate-depleted blackcurrant juice (PL). Blood samples were collected to measure NO3− and NO2− concentrations 2.5 h after consuming BJ or PL, with an additional sample taken 15 min before testing. All tests were conducted at the same time of day (± 2 h) under similar environmental conditions (temperature ∼22°C, relative humidity ∼50%) to compare the two experimental conditions. The last three visits were separated by a 7-day interval.

### 2.3 Supplement protocol

BJ and placebo PL were poured into opaque containers and prepared by a third party not involved in the research team. To ensure food safety throughout the process, the supplements were prepared in the laboratory. The sequence in which participants consumed each supplement was randomly assigned in a double-blind manner and coded by a third party outside the research team. The sequence of beetroot juice or placebo consumption was revealed to the researchers only after all data had been collected. Participants consumed one of the beverages—250 mL of BJ or 250 mL of PL—150 min before formal testing. The PL beverage, designed by nutrition experts to mimic the taste of commercial drinks, was prepared by dissolving 1 g of beetroot powder (Felicific Inc., New York, USA) and 9 g of blackcurrant powder (Han Traceability Biological Technology Co., Ltd., China) in 0.5 L of mineral water, with lemon juice added. Each serving of BJ (Biotta^<reg>(</reg>^; Biotta AG, Tägerwilen, Switzerland) and PL contained approximately 12.48 and 0.0005 mmol of NO3−, respectively, as determined by molecular absorption spectrophotometry (31). Since participants were aware of the study hypothesis, they were asked whether they had consumed BJ before (i.e., “Have you ever consumed BJ?”). Additionally, after each visit, they were asked to identify the type of supplement they believed they had consumed [i.e., “Which supplement do you think you consumed? (a) BJ; (b) PL; (c) Unsure”]. Participants were informed about potential side effects of BJ consumption, including gastrointestinal symptoms and red-colored stool and urine.

### 2.4 Maximal voluntary isometric contraction

The maximum voluntary isometric contraction (MVIC) of the upper and lower limbs was assessed to determine the individualized load required for subsequent experimental tests. A warm-up was performed before muscle strength and endurance measurements, consisting of a 5-min jog, 10 bodyweight squats, and 30 jumping jacks. The measurement protocol involved calibration of each device, followed by warm-up repetitions at 50, 70, and 90% of perceived maximal effort, performed for 4, 2, and 1 repetition(s), respectively, with a 2-min rest between loads. Each repetition was defined as achieving the target percentage of perceived maximal effort displayed in realtime on the screen, followed by relaxation. Subsequently, participants performed three 5-s MVICs, with a 2-min rest between each, and the highest value was recorded. Isometric handgrip (IH) was measured using an adjustable digital handgrip dynamometer (Electroni Hand Dynamometer, CAMRY, Model EH201R, Zhongshan Camry Electronic Co., Ltd., China). Participants stood upright with feet shoulder-width apart, arms relaxed, and performed the test by gripping the dynamometer as forcefully as possible with one hand. Isometric sit-up (ISU, during the ISU, only sEMG was recorded, in the absence of direct measurement methods for quantifying MVIC during sit-up movements) was tested with participants lying supine on a yoga mat. The abdomen, thighs, and calves formed a 90° angle, and the upper body was secured using two non-elastic straps during the ISU test. Isometric elbow flexion (IEF) and isometric knee extension (IKE) were tested using the isokinetic dynamometry system (iosmed 2000, D&R Ferstl GmbH, Hemau, Germany) under isometric mode with maximum resistance applied. IEF was assessed by setting the elbow joint angle to 90° angle. The equipment was calibrated according to system recommendations. Participants were seated with their back against the chair, shoulders and waist firmly strapped, and the angle was verified using a manual goniometer. The dominant hand gripped the handle, with the elbow joint aligned with the rotational center of the system, and the test was conducted. IKE was assessed by setting the knee joint angle to 20° angle. The equipment was adjusted according to system recommendations. Participants sat with their back against the chair, shoulders, waist, and the non-dominant thigh securely strapped, and the angle was confirmed using a manual goniometer. The dominant leg’s ankle was strapped to the device, with the knee joint aligned to the rotational center of the system, and the test was performed.

### 2.5 Exercise protocols

Participants first performed a MVIC test following a standardized warm-up. Subsequently, they performed three rounds of the ICET, with a one-minute rest between rounds. The ICET consisted of four consecutive subtests with no rest in between, requiring participants to maintain 70% of their respective MVIC until volitional fatigue (except ISU). The test commenced once the participant’s force output reached the target value. Fatigue was quantified as the point where the participant’s force output dropped below 95% of the target value for more than one second. Equipment calibration and participant positioning for the subtests were performed as described in Section “2.4 Maximal voluntary isometric contraction,” in the following order: (a) IEF test: After calibrating the isokinetic dynamometry system and securing the participant’s posture, resistance was set to 70% MVIC torque, and the test was conducted. (b) ISU test: Participants lay supine on a yoga mat with their abdomen, thighs, and calves forming a 90° angle. Hands were crossed behind the head. Abdominal contraction was performed to touch the thighs with the elbows, initiating the timer. Timing ended when the elbows lost contact with the thighs. (c) IH test: After securing the participant’s posture, resistance on the hand dynamometer was set to 70% MVIC, and the test was performed. (d) IKE test: Following calibration of the isokinetic dynamometry system and securing the participant’s posture, resistance was set to 70% MVIC torque, and the test was carried out. All joint angles were verified using a goniometer. Participants were instructed to maintain their force output as close as possible to the target force. Real-time force output was visualized on a computer screen, with the target force represented by a reference line. In addition to visual feedback, participants received verbal cues indicating whether their force output was “too high,” “too low,” or “on target.” The duration of contraction maintained above 95% of the target force was recorded.

### 2.6 Surface electromyography recording

Initially, the corresponding areas of the arms, abdomen, and thighs were shaved. After slight abrasion, the muscle regions were cleaned using alcohol. sEMG electrodes, configured with a 20 mm diameter bipolar silver chloride electrode, were placed on the muscle belly of the following muscles: the flexor digitorum superficialis (FDS), which is the most active muscle during hand gripping (32); the biceps brachii (BR); the rectus abdominis (RA); and the rectus femoris (RF). EMG signal acquisition was performed on the muscle belly region of these muscles. The electrodes were aligned parallel to the assumed direction of the muscle fibers, with an interelectrode distance of 20 mm. The EMG signals were amplified by a factor of 1.000, band-pass filtered (20–480 Hz), and sampled at 4 kHz using the WY-EMG system (Beijing Changfeng Technology Co., Ltd., China). Data analysis was conducted with the EmgServer software (Beijing Changfeng Technology Co., Ltd.). During the first experiment, non-fading markers were used to mark the positions of the EMG electrodes on the skin, ensuring consistent placement in subsequent trials. The EMG data from each muscle were normalized by calculating the root mean square (RMS) of the MVIC, and muscle activity during the tests was evaluated. This was expressed as a percentage of the RMS during maximum voluntary contraction (%MVIC).

### 2.7 Heart rate and ratings of perceived exertion

During the tests, heart rate values were recorded at the end of each round using a heart rate strap (Polar monitor, Kempele, Finland). Additionally, RPE were collected using the Borg CR10 scale. Participants were instructed to report their perceived exertion immediately after completing each round of the ICET.

### 2.8 Blood measurement

#### 2.8.1 Blood lactate

Blood lactate concentrations were measured using the Lactate Scout+ (EKF Diagnostics, Leipzig, Germany), with samples collected before and after the exercise tests. Samples were obtained after sterilizing the ring finger with 70% ethanol.

#### 2.8.2 Serum NO2− and NO3−

Venous blood was collected from the antecubital vein into 6 mL serum vacuum tubes (KWS, Hebei Kangweishi Medical Technology Co., Ltd., China). After natural coagulation for 60 min, the samples were centrifuged at 3,000 rpm for 10 min. The supernatant serum was then extracted and stored at −80°C until analysis. Serum nitrite/nitrate concentrations were measured using high-performance liquid chromatography (HPLC) as previously reported (33).

### 2.9 Statistical analysis

Normality of all data was confirmed using the Shapiro–Wilk test. A two-factor repeated-measures ANOVA (time × supplementation) was conducted to examine the effects of time points (ICET1, ICET2, ICET3), conditions (BJ vs. PL), and their interaction (time × condition) on exercise to fatigue (ETF), sEMG, heart rate, RPE, and blood lactate. When significant differences were detected, Bonferroni adjustments were applied for multiple comparisons. Paired-sample *t*-tests were used to compare plasma NO3−/NO2−, MVC, and ΔETF (ICET1-ICET3) between the two experimental conditions (BJ and PL). Effect sizes (ES) for ANOVA were calculated using partial eta-squared (), while Cohen’s *d* was used for paired *t*-tests. A confidence level of 0.05 was set for statistical significance. All statistical analyses were performed using SPSS version 26.0 (SPSS Inc., Chicago, IL, USA).

## 3 Results

### 3.1 Participants

Initially, 16 participants were recruited. One participant withdrew due to mycoplasma pneumonia infection during the study, and another dropped out after the first two visits due to a wrist injury unrelated to the experiment. Therefore, the final sample consisted of 14 participants. During the intervention, 8 participants reported red discoloration of feces/urine. To assess the blinding rate, participants were asked which beverage they believed they consumed in each trial. During BJ consumption, 43% believed they had taken the placebo, while 64% reported that they thought they had consumed BJ during the placebo trial.

### 3.2 Serum [NO3−] and [NO2−]

**FIGURE 3:**
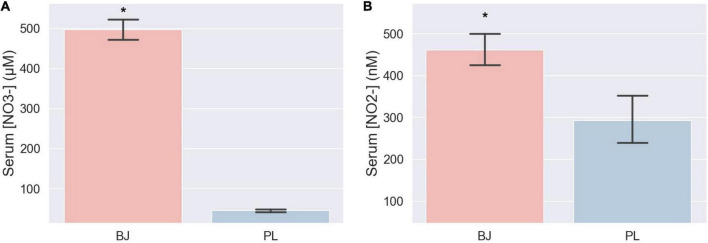
Serum NO3– concentration **(A)** and serum NO2– concentration **(B)** measured 2.5 h after supplementation showed significant differences between the two conditions (*P* < 0.05). *significantly different compared to PL.

Serum nitrate and nitrite levels in the BJ condition were 10.87-fold (*t* = 26.186, *P* < 0.001, *d* = 12.6, 95% CI [413.380, 487.777]; BJ: 496.24 ± 69.34 μM; PL: 45.66 ± 9.03 μM) and 1.57-fold (*t* = 6.136, *P* < 0.001, *d* = 4.66, 95% CI [109.031, 227.541]; BJ: 461.14 ± 99.58 nM; PL: 292.86 ± 153.04 nM) higher, respectively, compared to the PL condition (Figure 3).

### 3.3 Maximal isometric voluntary contraction

**FIGURE 4:**
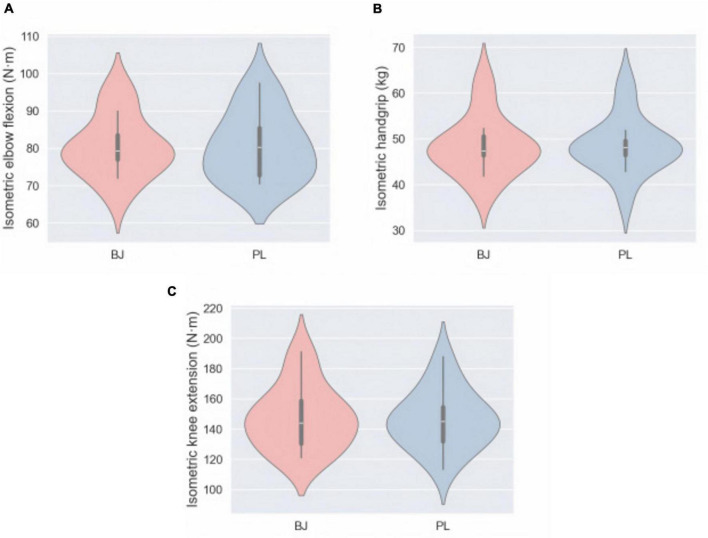
The mean (SD) values of MVIC, including its subcomponents IEF **(A)**, IH **(B)**, and IKE **(C)**, showed no statistical significance under the two conditions (*P* > 0.05). IEF, isometric elbow flexion; IH, isometric handgrip; IKE, isometric knee extension.

As shown in Figure 4, there were no significant differences in peak torque of the same muscle group’s MIVC across different conditions (*P* > 0.05).

### 3.4 Results of isometric circuit endurance test

**FIGURE 5:**
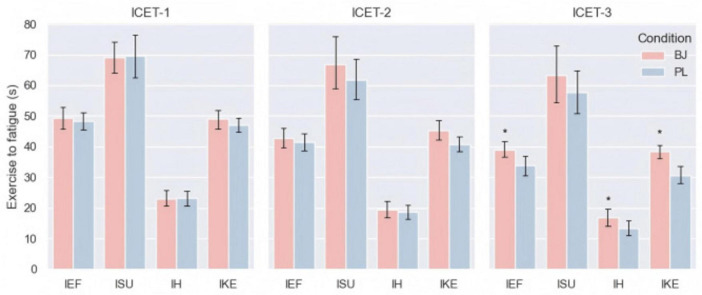
ETF at ICET-1, ICET-2 and ICET-3 in BJ and PL trials; Data are mean (SD); *significantly different compared to PL. ETF, exercise to fatigue.

The results of this section demonstrate the effects of BJ supplementation on four isometric endurance tests: IEF, ISU, IH and IKE (Figure 5).

#### 3.4.1 Elbow flexor performance

For the three rounds of IEF endurance, significant effects were observed for rounds (*F* = 57.907, *P* < 0.001, $\eta_{p}^{2}$ = 0.817) and the interaction between supplementation condition and rounds (*F* = 4.632, *P* = 0.019, $\eta_{p}^{2}$ = 0.262). Bonferroni correction revealed that in the third round, participants in the BJ condition sustained endurance longer than those in the PL condition (*P* = 0.012, 95% CI [1.340, 8.803]; BJ: 39.07 ± 1.87 s; PL: 34.00 ± 2.21 s). Additionally, there was a statistically significant difference in ΔETF between conditions (*t* = −2.520, *P* = 0.026, *d* = 0.674, 95% CI [−7.561, −0.582]; BJ: 10.36 ± 5.81 s; PL: 14.43 ± 6.25 s).

#### 3.4.2 Abdominal performance

For the three rounds of isometric abdominal endurance during the cyclic endurance test, no significant differences were observed for supplementation conditions (*F* = 0.717, *P* = 0.413, $\eta_{p}^{2}$ = 0.052), rounds (*F* = 3.745, *P* = 0.067, $\eta_{p}^{2}$ = 0.224), or the interaction between supplementation conditions and rounds (*F* = 1.290, *P* = 0.292, $\eta_{p}^{2}$ = 0.090). However, a significant difference in ΔETF was found between the BJ and PL conditions (*t* = −2.766, *P* = 0.022, *d* = 0.875, 95% CI [−10.361, −1.039]; BJ: 13.30 ± 7.53 s; PL: 19.00 ± 9.86 s).

#### 3.4.3 Forearm muscle performance

No significant main effect of supplementation condition was found (*F* = 1.959, *P* = 0.185, $\eta_{p}^{2}$ = 0.131). However, significant effects were observed for rounds (*F* = 47.942, *P* < 0.001, $\eta_{p}^{2}$ = 0.787) and the interaction between supplementation condition and rounds (*F* = 5.541, *P* = 0.010, $\eta_{p}^{2}$ = 0.299). Bonferroni correction indicated that during the third round of testing (*P* = 0.010, 95% CI [0.971, 5.886]; BJ: 16.93 ± 1.93 s; PL: 13.50 ± 1.83 s), participants in the BJ condition sustained IH endurance longer than those in the PL condition. Additionally, a significant difference in ΔETF was observed between the two conditions (*t* = −2.617, *P* = 0.021, *d* = 0.699, 95% CI [−6.389, −0.611]; BJ: 6.14 ± 3.90 s; PL: 9.64 ± 4.75 s).

#### 3.4.4 Knee extensor performance

Significant interaction effects between supplementation condition and rounds were observed across the three tests (*F* = 5.981, *P* = 0.007, $\eta_{p}^{2}$ = 0.315). Main effects were also found for supplementation condition (*F* = 7.411, *P* = 0.017, $\eta_{p}^{2}$ = 0.363) and rounds (*F* = 104.448, *P* < 0.001, $\eta_{p}^{2}$ = 0.889). Bonferroni correction revealed that during the third round (*P* = 0.001, 95% CI [3.922, 11.507]; BJ: 38.36 ± 1.67 s; PL: 30.64 ± 1.95 s), participants in the BJ condition exhibited significantly longer durations in the IKE test compared to the PL condition. Paired-sample *t*-tests further indicated a significant difference in ΔETF between conditions (*t* = −2.937, *P* = 0.012, *d* = 0.785, 95% CI [−10.165, −1.549]; BJ: 10.64 ± 6.16 s; PL: 16.50 ± 3.98 s).

### 3.5 Surface electromyography

**FIGURE 6:**
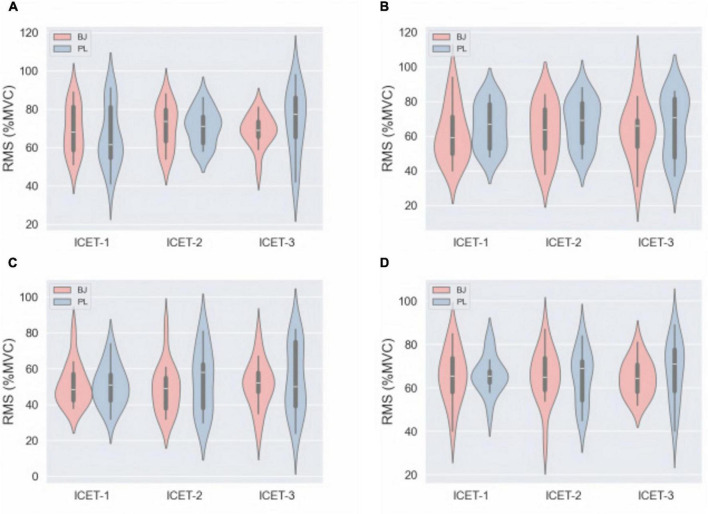
The mean (SD) values of RMS (%MVC), including its subcomponents IEF **(A)**, ISU **(B)**, IH **(C)**, and IKE **(D)**, showed no statistical significance under the two conditions (*P* > 0.05). IEF, isometric elbow flexion, IH, isometric handgrip, ISU, isometric sit-up, IKE, isometric knee extension.

No significant differences in RMS values were observed between the BJ and PL conditions across the four muscle groups during the three rounds of ICET (*P* > 0.05) (Figure 6).

### 3.6 RPE, HR and blood lactate

Significant main effects of rounds were observed for RPE, HR, and blood lactate at all time points (*P* < 0.001; see Supplementary Table 1). No significant effects of supplementation condition were detected (HR: *P* = 0.654; RPE: *P* = 0.763; BL: *P* = 0.085), nor were there any significant interaction effects between supplementation condition and rounds (HR: *P* = 0.598; RPE: *P* = 0.431; BL: *P* = 0.660).

## 4 Discussion

This study aimed to investigate the acute effects of BJ supplementation on MIVC, single-bout, and intermittent isometric endurance in different muscle groups. However, contrary to our hypothesis, the effects of BJ were observed only in certain muscle groups during the ICET-3. These findings suggest that acute nitrate supplementation does not improve MIVC or single-bout isometric endurance across muscle groups in collegiate bodybuilders but may enhance endurance by optimizing recovery during intermittent exercise.

In this study, acute ingestion of 250 mL BJ (∼12.48 mmol NO3−) did not produce statistically significant effects on the MVIC of forearm muscles, elbow flexors, or knee extensors in young bodybuilders. Although no prior studies have specifically examined the effects of beetroot juice supplementation on the MVIC of elbow flexors, our findings align with previous research evaluating the maximal isometric strength of other muscle groups (34–36). Specifically, the study by Lopez-Samanes et al. (37) demonstrated that acute ingestion of 140 mL beetroot juice (∼12.8 mmol NO3−) had no effect on IH strength. Similarly, Jonvik et al. (35) reported that six days of beetroot juice supplementation did not improve maximal isometric knee extensor strength. However, a few studies have reported contrasting results (24, 38). Notably, studies inconsistent with our findings often employed multi-joint, multi-muscle actions to test maximal voluntary isometric strength, such as mid-thigh isometric pulls or isometric box squats in a clean pull position. This suggests that nitrate supplementation may confer performance benefits when larger muscle groups are engaged (38). In contrast, the present study utilized more localized and isolated single-joint tests. This methodological difference may partly explain the discrepancies in findings. Future research should further explore the effects of nitrate supplementation on maximal strength performance across different exercise modalities.

It has been reported that bodybuilders experience significant muscle fatigue during competitions due to prolonged static contractions required for posing (2). Thus, accelerating the recovery of isometric endurance during short breaks between rounds or poses may be critical for maintaining well-defined muscle contours and overall performance. To the best of our knowledge, this is the first study to evaluate the effects of dietary NO3− supplementation on upper and lower limb cyclic isometric endurance in bodybuilders. Our findings indicate that NO3− supplementation can enhance isometric endurance. However, these results partially contradict some previous studies (39–41). While those studies did not show improvements in isometric endurance, they reported attenuation of strength loss following fatiguing exercise (39, 40). The discrepancies in results may be attributed to differences in exercise intensity and protocol. Previous studies used isometric endurance tests with rhythmic contraction-relaxation cycles at moderate intensity (40%–50% 1RM), unlike the higher-intensity (70% 1RM), sustained-to-failure protocol applied in this study. Animal and human studies suggest that NO3− supplementation enhances physiological and performance responses of type II muscle fibers (42), which are more actively recruited as exercise intensity increases (43). This may explain why our study found no significant differences in abdominal muscle isometric endurance. One reason is the higher proportion of type I muscle fibers in core abdominal muscles (44). Another reason may be the relatively lower load intensity in the ISU test. Although this study did not specify the load for the ISU test, its longer duration compared to the other three subtests suggests lower intensity, insufficient to recruit type II fibers effectively. In skeletal muscle, intramuscular pressure induced by muscle fiber contraction increases with force. As contraction intensity continues to rise, this pressure may become high enough to restrict or even completely occlude blood flow (45). Such blood flow restriction can limit oxygen delivery to muscles during high-intensity rhythmic exercise (46). Additionally, under conditions of acidosis and hypoxia, the reduction of NO2− to NO is enhanced (47). For example, in a study by Porcelli et al. (48), five days of NO3− supplementation (8.2 mmol/day) improved the number of repetitions performed during a rhythmic IKE test at high intensity (75% 1RM). Sustained isometric contractions are reported to cause greater blood flow restriction than dynamic contractions (7, 49), and the vasodilatory effects of NO3− on vascular smooth muscle (13) may be amplified under ischemic conditions (50). Papadopoulos et al. (51) observed an 8.5% increase in endurance time during a low-intensity (30% 1RM) sustained forearm isometric test following arterial occlusion using a cuff. Similarly, Poredos et al. (34) reported improvements in isometric endurance during moderate-intensity (50% 1RM) sustained knee extension tests. Therefore, we hypothesize that NO3− may exert ergogenic effects under conditions of severe blood flow restriction, such as rhythmic high-intensity or sustained moderate-to-high-intensity contractions. Further research is needed to determine the degree of blood flow restriction required to elicit the ergogenic effects of NO3− and whether our findings can be replicated in other sports or athletic populations requiring isometric endurance.

Following NO3− supplementation, no statistically significant differences were observed in the isometric endurance performance of different muscle groups between the first and second rounds, whereas the ΔETF was significant, contradicting the initial hypothesis. This suggests that the effect of beetroot juice on performance may be influenced by the degree of muscle fatigue or the rate of recovery during rest intervals. Previous animal studies reported that during the early stages of fatigue-induced contraction protocols, mouse muscle cell contractile function and Ca2+ handling remained unchanged. However, in later stages, task failure time was extended due to better maintenance of muscle cell contractile force, Ca2+ sensitivity, and Ca2+ pumping (52). Similarly, Tillin et al. (53) reported that NO3− supplementation mitigated muscle fatigue and more effectively enhanced the contractile force of fatigued skeletal muscle. Additionally, discrepancies in prior research findings may stem from differences in participant training levels. Highly trained individuals may exhibit greater NO synthesis efficiency (54), and in bodybuilders, hypertrophied muscle fibers may reduce capillary density, increasing the oxygen or blood flow demand of active muscles (55), thereby diminishing the benefits of NO3− supplementation (56). Future research should explore the effects of NO3− supplementation across individuals with varying training levels, muscle fiber compositions, and exercise modalities while elucidating the underlying physiological mechanisms.

In this study, sEMG data indicated that acute NO3− supplementation did not significantly alter muscle electrical activity patterns, consistent with findings from previous studies (48, 57). This suggests that NO3− supplementation may enhance muscle contraction efficiency through mechanisms such as increased mitochondrial efficiency (58), improved hemodynamics (26), or optimized muscle contractile mechanisms (12), rather than directly influencing neuromuscular activation. Additionally, the participants in this study were well-trained bodybuilders, who likely already possess highly efficient neuromuscular activation patterns (59). Therefore, in highly trained individuals, NO3− supplementation may exert its effects primarily by optimizing other potential mechanisms rather than altering already well-adapted neuromuscular activation patterns. Future studies are needed to evaluate how NO3− supplementation modulates excitation-contraction coupling in skeletal muscle.

## 5 Conclusion

This study demonstrated that acute BJ supplementation had no significant effect on the MIVC of collegiate bodybuilders. However, certain muscle groups showed improved endurance during high-intensity intermittent isometric endurance tests. This suggests that nitrate may possibly enhance endurance by optimizing recovery during intervals, rather than directly increasing strength. The effect of BJ on type II muscle fibers may be more pronounced under high-intensity conditions, while its impact on muscle groups with a higher proportion of type I fibers appears limited. Furthermore, BJ did not significantly alter muscle electrical activity, potentially enhancing muscle contraction efficiency through mechanisms such as increased mitochondrial efficiency, improved hemodynamics, or optimized muscle contractile processes. Future research should further investigate the effects of nitrate supplementation on individuals with varying training levels, training intensities and recovery protocols to better understand its underlying physiological mechanisms.

## Data Availability

The original contributions presented in this study are included in this article/Supplementary material, further inquiries can be directed to the corresponding author.
