# Supplementary material for: Effects of dietary nitrate supplementation on isometric performance and physiological responses in college bodybuilders: a randomized, double-blind, crossover study
Source: Front Nutr. 2025 May 8;12:1576712. doi: 10.3389/fnut.2025.1576712 (PMC12095002; doi:10.3389/fnut.2025.1576712)
Supplement: Supplementary file 1 [file Table_1.docx]

Supplementary Material

# Supplementary Tables

**Supplementary Table 1. Heart rate, RPE, and blood lactate concentration results before and after the test.**

|  | **HR（bpm）** | | **RPE** | | **BL (mmol/L)** | |
| --- | --- | --- | --- | --- | --- | --- |
|  | **BJ** | **PL** | **BJ** | **PL** | **BJ** | **PL** |
| ****Pre-test**** | 71 ± 8 | 70 ± 5 | - | - | 3.70 ± 3.14 | 4.81 ± 3.78 |
| **ICET-1** | 112 ± 13 | 108 ± 14 | 6.11 ± 1.98 | 6.36 ± 1.56 | - | - |
| **ICET-2** | 115 ± 12 | 115 ± 17 | 7.21 ± 1.57 | 7.07 ± 1.05 | - | - |
| **ICET-3** | 117 ± 12 | 117 ± 14 | 7.50 ± 1.51 | 7.75 ± 1.89 | 13.84 ± 6.54 | 15.87 ± 6.28 |

Abbreviations:HR, heart rate; RPE, ratings of perceived exertion; BL, blood lactate; BJ, beetroot juice; PL, placebo; ICET,isometric circuit endurance test.
